# Pediatric aseptic lower leg fracture nonunion

**DOI:** 10.1007/s00068-020-01556-1

**Published:** 2020-12-02

**Authors:** Christian von Rüden, Sven-Oliver Dietz, Peter Schmittenbecher, Francisco F. Fernandez, Justus Lieber, Björn Wilkens, Matthias Rüger, Dorien Schneidmueller

**Affiliations:** 1 Department of Trauma Surgery, BG Unfallklinik Murnau, Professor Küntscher Str. 8, 82418 Murnau, Germany; 2 Department of Trauma Surgery, Sports Orthopaedics and Pediatric Traumatology, Klinikum Garmisch-Partenkirchen, Garmisch-Partenkirchen, Germany; 3 Institute for Biomechanics, Paracelsus Medical University, Salzburg, Austria; 4 Department of Orthopaedics and Traumatology, University Medical Center, Johannes Gutenberg-University, Mainz, Germany; 5 Department of Pediatric Surgery, Städtisches Klinikum Karlsruhe, Karlsruhe, Germany; 6 Department of Orthopaedic Surgery, Olgahospital, Klinikum Stuttgart, Stuttgart, Germany; 7 Department of Pediatric Surgery and Pediatric Urology, University Children’s Hospital of Tuebingen, Tübingen, Germany; 8 Department of Pediatric Surgery and Pediatric Urology, Krankenhaus St. Elisabeth und St. Barbara, Halle/Saale, Germany; 9 Pediatric Orthopaedics and Traumatology, University Children’s Hospital Zurich, Zurich, Switzerland

**Keywords:** Nonunion, Pseudarthrosis, Lower leg, Tibia, Fibula, Pediatric, Children, Adolescents

## Abstract

**Purpose:**

Lower leg nonunion in pediatric patients is a rarity. Therefore, eight European pediatric trauma units retrospectively analyzed all patients younger than 18 years suffering lower leg fractures resulting in aseptic nonunion.

**Methods:**

Thirteen children and adolescents less than 18 years old (2 girls and 11 boys) diagnosed with aseptic nonunion of the tibia and/or fibula were evaluated. In all patients, epidemiological data, mechanism of injury, fracture configuration, and the initial treatment concept were assessed, and the entire medical case documentation was observed. Furthermore, potential causes of nonunion development were evaluated.

**Results:**

The mean age of patients was 12.3 years with the youngest patient being seven and the oldest being 17 years old. Open fractures were found in six out of thirteen patients (46%). Nonunion was hypertrophic in ten and oligotrophic in three patients. Mean range of time to nonunion occurrence was 7.3 ± 4.6 months. Nonunion healing resulting in complete metal removal was found in 12 out of 13 patients (92%), only in one case of a misinterpreted CPT type II osseous consolidation could not be found during the observation period. Mean range of time between surgical nonunion revision and osseous healing was 7.3 months as well.

**Conclusion:**

If treatment principles of the growing skeleton are followed consistently, aseptic nonunion of the lower leg remains a rare complication in children and adolescents. Factors influencing the risk of fracture nonunion development include patient’s age, extended soft tissue damage, relevant bone loss, and inadequate initial treatment.

## Introduction

Fracture nonunion is difficult to treat and represents a challenge for the treating surgeon as well as it is a physical and psychological burden for the young patient. There is a general consensus that regardless of patients’ age, fracture healing depends on a number of factors such as the complexity of the fracture, blood supply to the fracture site, bone stability, existing inflammation and existing preconditions [1–3]. The management of fracture nonunion in long bones remains a hot topic. In adults, the incidence of fracture nonunion ranges from 15% for Gustilo grade II fractures to over 80% for Gustilo grade IIIB fractures [4–6]. Although reliable data is not available for children and adolescents, the microvascular circulation and osteoblast activity are considered to be essential biological factors independent of patients’ age [7]. In addition, the organization of the fracture hematoma and preparation phases and especially the cellular organization phase play an important role [8]. In pediatric fractures, these biological factors compete and complete each other in fracture healing, so that overall development of nonunion is very rare [9, 10]. In this respect, this topic receives little or no attention in the relevant literature on pediatric traumatology [10, 11]. Sporadic lower leg fracture nonunion is reported in individual case series in adolescents with adult-like physique following various different surgical treatment concepts [12–17]. Most of these cases involve adolescents over 10 years of age [18]. In the existing literature, lower leg nonunion in children mainly affects complicated fractures, which are accompanied by a clear bone defect as well as massive soft tissue injuries and therefore also inflammation [19–22]. In several other cases, open reduction and internal fixation was performed. Even in the presence of the above-mentioned unfavorable factors it is claimed that in most cases of fracture nonunion in children and adolescents it is a consequence of incorrect treatment [22–24]. Interestingly, publications on the conservative treatment of closed lower leg fractures in children and adolescents do not address nonunion development [9]. It may be assumed that a certain number of unreported cases exist and that the rate of lower leg fracture nonunion in children and adolescents is higher than described in the literature.

This article provides a basic overview on factors leading to aseptic lower leg fracture nonunion in children and adolescents as well as a series of cases including current treatment concepts.

## Patients and methods

Eight European pediatric trauma units retrospectively analyzed all patients with open epiphyseal plates with lower leg fractures resulting in aseptic nonunion. Since lower leg nonunion in pediatric patients is very rare, there was no limitation as to how old the cases were. Thirteen children and adolescents less than 18 years old (2 girls and 11 boys) diagnosed with aseptic nonunion of the tibia and/or fibula were evaluated. Patients with nonunion due to initially diagnosed congenital causes or to tumors as well as patients with nonunion following infection-related pathological fractures were excluded from the study. In all patients, gender, age, mechanism of injury, fracture configuration, and initial treatment concepts were assessed. Furthermore, range of time between initial fracture treatment and occurrence of nonunion was evaluated. Preoperative, intraoperative, and postoperative anterior–posterior (AP) and lateral radiographs as well as the entire medical case documentation were observed. In each case, radiographic findings were evaluated, nonunion was classified according to the system described by Weber and Cech [25], and potential causes of nonunion development were evaluated.

## Results

**Fig. 1:**
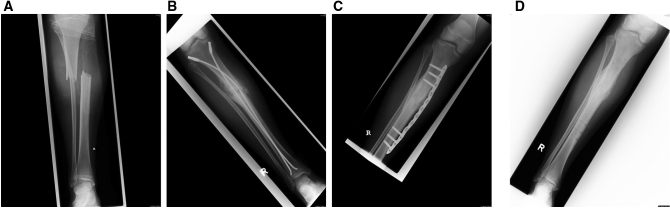
**a** Fourteen-year-old girl had an accident as a passenger on a scooter resulting in a displaced first degree open transverse tibial shaft fracture. **b** Six months after closed reduction and internal fixation using two Elastic Stable Intramedullary Nails (ESIN) including locking with end caps, hypertrophic aseptic tibial shaft nonunion and subsequent nail breakage was assessed. **c** Surgical revision was performed including debridement of the nonunion site, internal compression plate fixation and autologous cancellous bone grafting from the surrounding area. **d** Six weeks after revision surgery, radiological follow-up demonstrated osseous healing resulting in complete metal removal

**Fig. 2:**
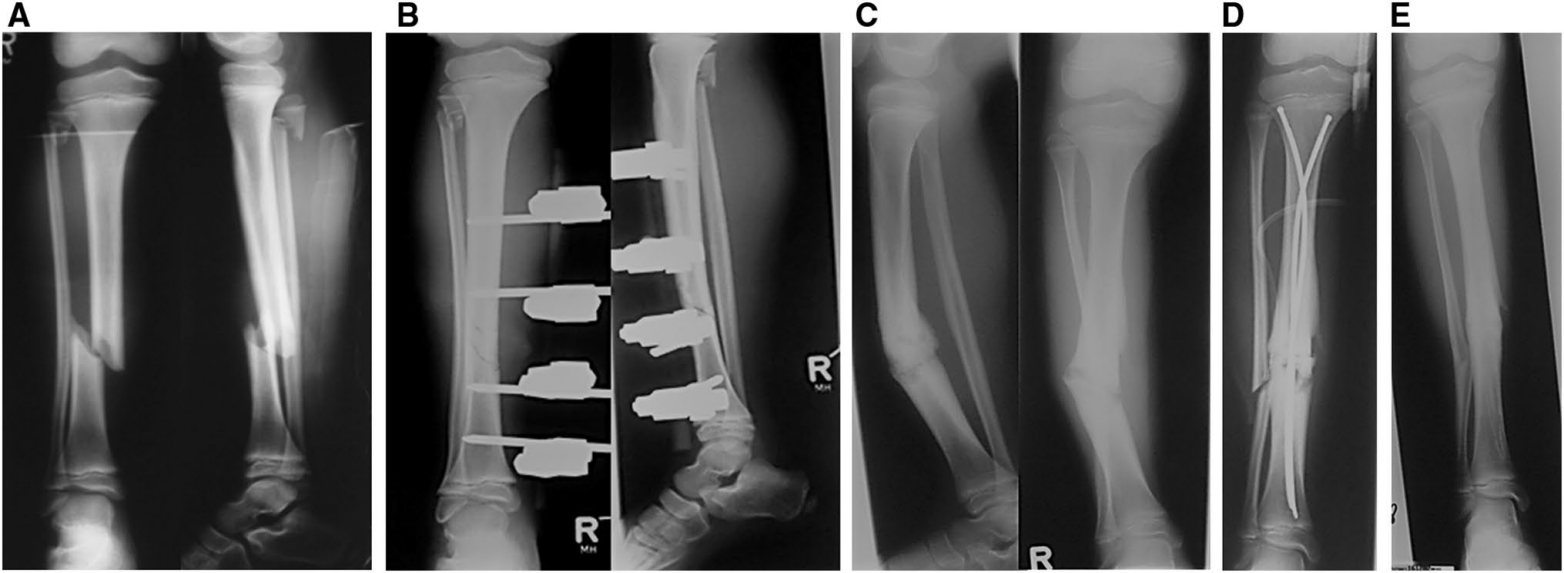
**a** A bike accident in a 10-year-old boy resulted in Gustilo grade II open tibial shaft fracture. **b** Index surgery was performed with closed reduction and external fixation. The external fixation was removed after ten weeks. In the further clinical course pain occurred during full weight bearing. **c** Radiological follow-up demonstrated aseptic tibial shaft nonunion accompanied by varus axis deviation. **d** Revision surgery was performed with reaming of the nonunion site, fibula osteotomy, and closed tibial shaft axis correction using two ESIN. **e** Bony healing was assessed 16 weeks after surgical revision leading to complete metal removal

**Fig. 3:**
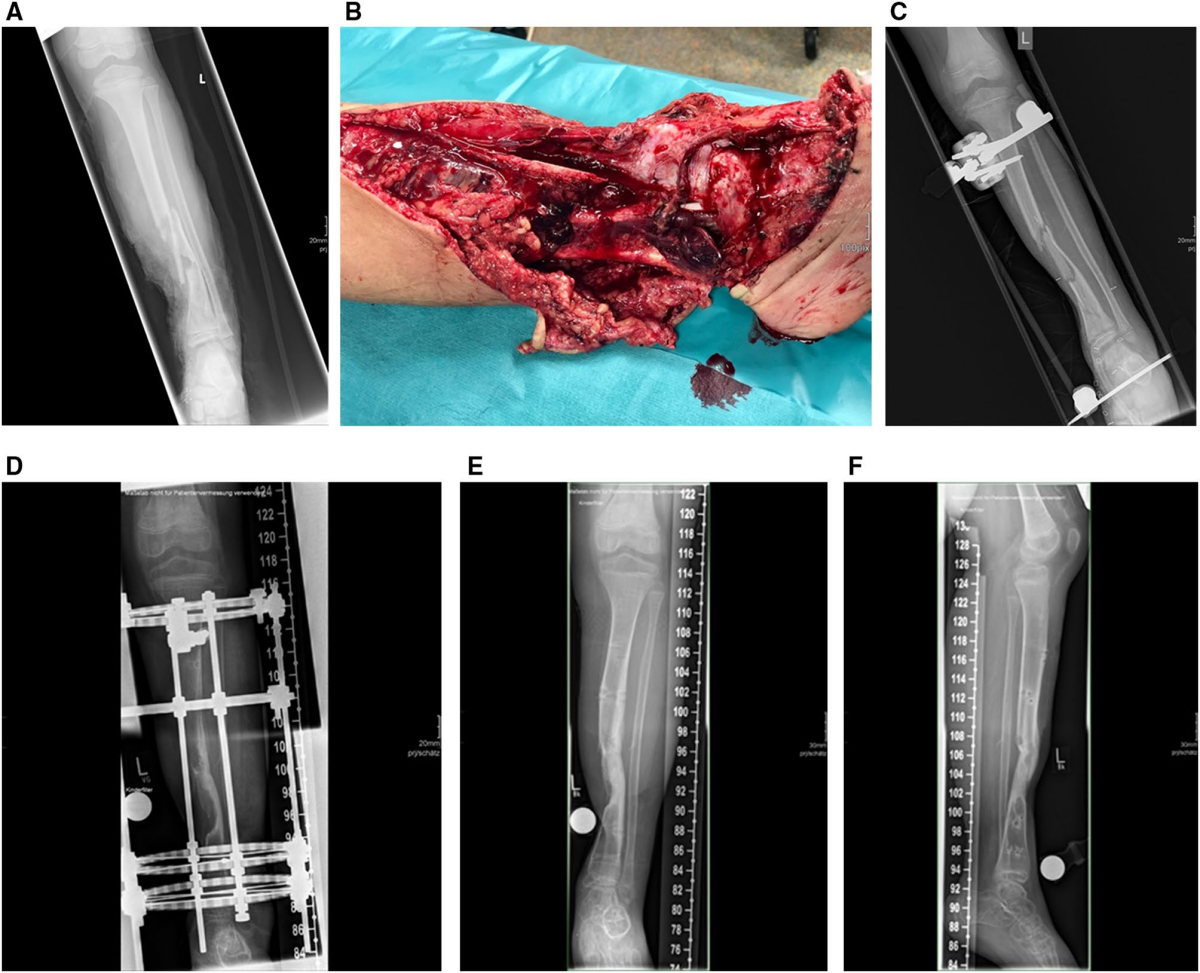
**a**, **b** Eight-year-old boy rolled over by a school bus resulting in Gustilo grade IIIB open multi-fragmentary tibial fracture. **c** Primary treatment including closed fracture reduction and stabilization by means of an external fixator. Nonunion was defined after lack of bone healing for 6 months. **d** Surgical revision with construction of an Ilizarov ring fixator enabled loading. **e**, **f** Osseous healing of the defect was found radiologically and clinically 12 months after surgical revision

An overview of patient data and course of injury in each case is provided in Table 1. The mean age was 11.5 ± 3.5 years with the youngest patient being seven and the oldest being 17 years old. In regards to age distribution, none of the patients was younger than 7 years. Mean age of patients with nonunion following open fracture was 10.7 ± 2.3 years and in patients with nonunion following closed fractures 10.7 ± 3.7 years. Mechanism of injury was a bicycle accident in five patients, a traffic accident as pedestrian in three patients, a scooter accident in three patients, and a collision during soccer play in another two patients. According to the nonunion classification system provided by Weber and Cech [25], nonunion was hypertrophic in 10 cases and oligotrophic in three cases. According to the Gustilo and Anderson classification [26], open fractures were found in six out of thirteen patients (46%). Initial conservative treatment was performed in three closed fractures. Closed reduction and external fixation was used in three open fractures and in another case of a closed fracture. In three cases, initial treatment included closed reduction and intramedullary nailing. In one case, closed reduction and elastic stable intramedullary nailing and in one other case combined screw and Kirschner wire fixation was performed. In the remaining case, open reduction and internal fixation (ORIF) using a small fragment plate was done. The mean range of time to nonunion was 7.3 ± 4.6 months (mean ± standard deviation). The potential reason for development of nonunion in each patient is provided in Table 1. Definitive surgical revision included locking compression plating (LCP) combined with autologous cancellous bone grafting in six patients (Fig. 1a–d), reamed tibial exchange nailing in three patients, elastic stable intramedullary nailing in one patient (Fig. 2a–e) and external fixation methods in two patients (Fig. 3a–f) [27, 28]. In one of those three patients with external fixation tibial nonunion initially was considered to be aseptic. In the further clinical course when surgical revision failed it was diagnosed as a congenital pseudarthrosis of the tibia (CPT) type II according to the Crawford classification instead of a common tibial shaft nonunion [29]. Nonunion healing resulting in complete metal removal was achieved in 12 out of 13 patients (92%), only in the case of CPT type II no osseous consolidation was obtained in the observation period. The mean range of time between nonunion revision and osseous healing was 7.3 months.

**Table 1:** Overview of patient data and clinical course

No	Gender	Age [years]	Mechanism of injury	Fracture type	Soft tissue damage	Initial therapy	Range of time to nonunion [months]	Type of nonunion according to Weber and Cech [25]	Potential reason for nonunion development	Number of revisions before transfer to another hospital	Surgical revision	Range of time to osseous healing [months]	Metal removal
1	Male	8	Bicycle accident	Tibial shaft fracture	Closed	Conservative	6	Hypertrophic	No obvious reason for nobunion	–	LCP + autologous cancellous bone from ipsilateral tibial head	4	Yes
2	Female	7	Bicycle accident	Salter II proximal tibial fracture	Closed	Conservative	4	Oligotrophic	Unstable situation due to initial conservative treatment	1	External fixation + Kirschner wire fixation + autologous cancellous bone from ipsilateral calcaneus	3	Yes
3	Male	9	Bicycle accident	Distal tibial shaft fracture	Closed	Conservative	6	Hypertrophic	No obvious reason for nonunion	–	LCP + autologous cancellous bone from ipsilateral tibial head	3	Yes
4	Male	13	Traffic accident as pedestrian	Tibial shaft fracture	Open (Gustilo gade I)	External fixator	8	Hypertrophic	No obvious reason for nonunion	–	LCP + autologous cancellous bone from local tissues	6	Yes
5	Male	16	Scooter accident	Tibial and fibula shaft fracture	Closed	Unreamed tibia nail	4	Hypertrophic	Unreamed nail with too small diameter; fibula blocking	–	Reamed tibia exchange nailing + fibula osteotomy	2	Yes
6	Male	17	Scooter accident	Tibial and fibula shaft fracture	Closed	Reamed tibia naiH	8	Hypertrophic	No obvious reason for nonunion	–	Reamed tibia exchange nailing	4	Yes
7	Male	16	Bicycle accident	Tibial and fibula shaft fracture	Open (Gustilo gade I)	Reamed tibia nail	4	Hypertrophic	Fibula blocking; ORIF + cerclage wiring	1	Reamed tibia exchange nailing + auxiliary plate + fibula osteotomy	3	Yes
8	Female	14	Scooter accident	Tibial shaft fracture	Open (Gustilo gade I)	2 × ESIN 3.5 mm with end caps	6	Hypertrophic	Too rigid fixiation due to the additional use of end caps in a transverse fracture	–	LCP + autologous cancellous bone from local tissues	1	Yes
9	Male	13	Soccer collision	Fibula shaft and Salter and Harris II tibial fracture	Closed	Tibia: screw + 2 Kirschner wires Fibula: conservative without any reduction	5	Hypertrophic fibula nonunion	Complete fibula shaft displacement including shortening resuling in potential instability	1	LCP + reaming debris + autologous cancellous bone from local tissues	9	Yes
10	Male	10	Soccer collision	Tibial and fibula shaft fracture	Closed	External fixator	10	Oligotrophic CPT type II	Not seen CPT	2	Segmental tibial osteotomy, nonunion resection, fibula osteotomy, Taylor Spatial Frame (TSF), intramedullary rodding of the tibia, ESIN of the fibula (Catanzano 2018)	–	No
11	Male	9	Traffic accident as pedestrian	Distal tibial fracture	Open (Gustilo grade II)	ORIF small fragment plate	22	Hypertrophic	No obvious reason for nonunion	3	Segmental tibial and fibula osteotomy, nonunion resection, LCP + autologous cancellous bone from local tissues	25	Yes
12	Male	10	Bicycle accident	Tibial and fibula shaft fracture	Open (Gustilo grade II)	External fixator	6	Hypertrophic	No obvious reason for nobunion	–	Reaming of the nonunion site, fibula osteotomy, closed tibial shaft axis correction using two ESINs	16	Yes
13	Male	8	Rollover trauma as pedestrian	Multifragmentary tibial shaft fracture	Open (Gustilo grade IIIB)	External fixator	6	Oligotrophic	Insufficient initial external fixation; open fracture with extended soft tissue damage	–	Nonunion resection, Ilizarov ring fixator	12	Yes

## Discussion

Fracture nonunion is a rare complication in fractures of long bones where the growth plates are not yet closed. In children under 12 years of age this complication is seen even less frequently [30]. Age is considered to be one of the main factors for developing of nonunion in adolescents. From 12 years upwards, delayed fracture union or nonunion was seen in similar orders of magnitude comparable to those in adults [15, 31]. Furthermore, it could be demonstrated that delayed union and nonunion formation did not occur below the age of six years and that there was a significant difference in healing rates before and after this age. In further investigations it was observed that the majority of nonunion occurred in adolescents between 12 and 16 years of age [9, 32]. In our patient collective mean age was 12 years with the majority of patients older than 12 years. In this respect, our results confirm the current literature.

The rate of open fractures in the current study was almost 50%, which represents another unfavorable factor for developing a lower leg nonunion [15]. Besides, a trend towards a more frequent occurrence of nonunion in the lower extremity compared to the upper extremity was seen [9]. Regarding the question of the influence of closed or open fractures, this factor seems to have significantly more importance in adolescents than in adults [33]. The risk of nonunion formation in children and adolescents with open fractures appears to be significantly higher than in closed fractures. After growth completion in closed epiphyseal growth plates, biomechanical reasons for nonunion formation are increasingly coming to the fore. While in children with conservatively treated closed tibial fractures the development of nonunion only plays a subordinate role, in adolescents with an adult-like physique and closed growth plates the rate of nonunion appears to be of the same order of magnitude as in adults. However, in children with open tibial fractures, nonunion rates do not appear to be of the same order of magnitude as in adults, especially not in children with relevant segmental bone defects and excessive skin-soft tissue injuries if the fracture is correctly treated [22, 24, 32, 34, 35]. One reason might be that the immature skeleton has an enormous potential to form new bone when the biologic and mechanical environment is favorable [11].

Undoubtedly, some genetic and cytogenetic changes necessary for fracture healing are associated with the development of fracture nonunion, although many aspects of the pathogens are still unclear at a molecular level. A number of different new approaches to understand the different genes and cytokines involved in the early identification of nonunion could be helpful in the prevention and treatment of nonunion. However, there are different causes for the different types of nonunion, septic and aseptic or atrophic, oligotrophic, and hypertrophic. Genetic variations and external risk factors have been identified as the main causes of abnormal cytokine expression. There is evidence that some cytokine changes are more likely to be caused by genetic mutations than by external risk factors [8]. However, there is still a lack of evidence in this regard through validation in animal and human studies, as well as for the interaction and coordination between gene variations and external risk factors and for the correlation between different cytokines in the development of fracture nonunion.

A further influencing factor is that in children open reduction and internal fixation has a negative effect on fracture healing if the fixation material is incorrectly selected and interferes with fracture healing [9]. Negatively influencing factors such as the selection of the wrong osteosynthesis, inadequate fracture fixation, or infection play a decisive role in the development of tibial nonunion after surgery. This is confirmed by the data of the current study. Indications for surgical therapy should therefore be considered and limited open procedures should be preferred in order to not promote delayed fracture healing. The principles of a modern fixation procedure must be taken into account for children, too. Additionally, similar to nonunion in adults, the interposition of autologous bone graft in the form of an autologous bone chip or cancellous bone also represents a therapeutic option for nonunion treatment in children and adolescents [36]. Various bone grafts can be used, such as vascularized and non-vascularized chips with or without structural support for the healing bone, or reaming debris [37, 38]. Although the stimulating effect on bone healing is undisputed, unfavorable side effects and risks such as the removal morbidity, the risk of infection, or the risk of insufficient healing of the graft may occur [39].

During the collection of data, an interesting special case was observed. During course of treatment the patient was re-diagnosed with a CPT instead of a common tibial shaft nonunion [40]. In this case, the morphology of the fibula could have made one skeptical. One may learn from this case that it is worthwhile considering the possibility of a CPT. Besides, gradual deformity correction by distraction osteogenesis is considered to be a conventional surgical management strategy not only in cases of CPT, but for lower leg fracture nonunion in general [41]. In cases with significant multi-planar deformities, it requires careful pre-operative planning and execution, which involves long periods of “dynamic” phases of the Ilizarov method [42–44].

Finally, another decisive factor in the treatment of pediatric tibial fractures is the correct aftercare treatment. In the growing skeleton, different basic considerations than in adults are necessary. The age-related activity level and compliance of the patient must be taken into account as well as the growth potential of the bone. Therefore, errors in aftercare treatment as well as in the indication for too early or too late metal removal can also lead to delayed union or nonunion [45, 46].

## Conclusion

The occurrence of delayed fracture union or nonunion of the lower leg in children is very rare, but in adolescents with adult-like physique it is of the same order of magnitude as in adults. The decisive factor is a therapy appropriate to the age group, which includes a conservative or surgical approach as well as consistent follow-up treatment. Essential factors such as loss of bone substance or skin and soft tissue defects may lead to the development of nonunion despite careful therapy. Nevertheless, if the treatment principles of the growing skeleton are followed consistently, aseptic lower leg nonunion remains a rare complication in fracture management of children and adolescents.

## Data Availability

The datasets analyzed during the current work are available from the corresponding author upon reasonable request.
